# The anti-tumor efficacy of 2-deoxyglucose and D-allose are enhanced with p38 inhibition in pancreatic and ovarian cell lines

**DOI:** 10.1186/s13046-015-0147-4

**Published:** 2015-04-01

**Authors:** Scott W Malm, Neale T Hanke, Alexander Gill, Liliana Carbajal, Amanda F Baker

**Affiliations:** College of Pharmacy, University of Arizona, Tucson, Arizona USA; College of Medicine, University of Arizona Cancer Center, Tucson, Arizona USA

**Keywords:** 2-deoxy-glucose, D-allose, Hypoxia inducible factor 1α, Pancreatic cancer, Ovarian cancer, p38 mitogen activated protein kinase

## Abstract

**Purpose:**

The anti-tumor activity of glucose analogs 2-deoxy-glucose (2-DG) and D-allose was investigated alone or in combination with p38 mitogen-activated protein kinase (MAPK) inhibitor SB202190 or platinum analogs as a strategy to pharmacologically target glycolytic tumor phenotypes.

**Methods:**

Hypoxia inducible factor-1 alpha (HIF-1α) protein accumulation in pancreatic cell lines treated with SB202190 alone and in combination with glucose analogs was analyzed by Western blot. HIF-1α transcriptional activity was measured in MIA PaCa-2 cells stably transfected with a hypoxia response element luciferase reporter following treatment with glucose analogs alone, and in combination with SB202190. Induction of cleaved poly(ADP-ribose) polymerase (PARP) was measured by Western blot in the MIA PaCa-2 cells. *In vitro* anti-proliferative activity of 2-DG and D-allose alone, or in combination with oxaliplatin (pancreatic cell lines), cisplatin (ovarian cell lines), or with SB202190 were investigated using the MTT assay.

**Results:**

SB202190 decreased HIF-1α protein accumulation and transcriptional activity. 2-DG demonstrated greater anti-proliferative activity than D-allose. Pre-treatment with SB202190 enhanced activity of both 2-DG and D-allose in MIA PaCa-2, BxPC-3, ASPC-1, and SK-OV-3 cells. The combination of D-allose and platinum agents was additive to moderately synergistic in all but the OVCAR-3 and HEY cells. SB202190 pre-treatment further enhanced activity of D-allose and 2-DG with platinum agents in most cell lines investigated.

**Conclusions:**

SB202190 induced sensitization of tumor cells to 2-DG and D-allose may be partially mediated by inhibition of HIF-1α activity. Combining glucose analogs and p38 MAPK inhibitors with chemotherapy may be an effective approach to target glycolytic tumor phenotypes.

## Background

Pancreatic cancer and ovarian cancer are aggressive, frequently chemotherapy resistant malignancies characterized by a high degree of molecular heterogeneity [1,2]. Both tumor types have been shown to be glycolytically active as evidenced by nuclear medicine imaging [3,4]. Targeting the metabolic phenotype of these tumors, rather than a specific genotype, is an attractive therapeutic strategy which may help overcome resistance to standard therapies.

Aerobic glycolysis, often referred to as the Warburg effect, is the conversion of glucose to lactic acid in the presence of oxygen and is one of the core hallmarks of both primary and metastatic cancers [5]. This adaptive metabolic mechanism is associated with a proliferative advantage and is closely linked with tumor invasiveness due to the increased production of lactic acid in the extracellular microenvironment [6]. Hypoxia-inducible transcription factor-1 alpha (HIF-1α) is the primary transcription factor that regulates the expression of genes essential for glycolysis including glucose transporter 1, hexokinase 1 and 2, and lactate dehydrogenase A (LDHA) [6]. HIF-1α message is continually expressed, but the protein is quickly ubiquitinated and degraded by the proteasome under normoxic conditions. However, constitutive expression of HIF-1α protein occurs in some tumor cells due to activation by growth factors, or mutations in Von-Hippel-Lindau (VHL), tumor protein 53, or phosphatase and tensin homolog [reviewed in [7]]. In addition, reactive oxygen species (ROS) can regulate HIF-1α transcription and translation via the phosphoinositide 3-kinase/protein kinase B (PI3K/AKT) and extracellular signal-regulated kinases (ERK) pathways resulting in HIF-1 α accumulation and transcriptional activity in normoxia and hypoxia [reviewed in [8]].

Inflammation is another hallmark of cancer and is a common component of the pancreatic [9,10] and ovarian tumor [11,12] microenvironment that has recently been shown to have an impact on tumor cell metabolism. Cancer associated fibroblasts in the tumor stroma secrete pro-inflammatory cytokines such as Tumor Necrosis Factor-alpha and Interleukin-17 that can induce aerobic glycolysis in adjacent tumor cells via upregulation of transcription factors including HIF-1α, avian myelocytomatosis virus mammalian homolog, and Nuclear Factor Kappa B [13,14]. This effect has been referred to as “metabolic symbiosis”. The p38 mitogen activated protein kinase (MAPK) pathway transduces signals in response to multiple inflammatory mediators and oxidative stress and has been shown to be a critical regulator of inflammation-associated tumorigenesis [2]. Nuclear expression of p38 MAPK and ERK 1/2 in pancreatic tumors is associated with shorter survival times [15]. Inhibitors of the p38 MAPK pathway are in pre-clinical and clinical development for cancer and inflammatory diseases [reviewed in [16]]. The biological effects of p38 MAPK inhibitors are highly tissue, context, and time dependent [17]. ERK 1/2 and p38 MAPK have both been implicated in the phosphorylation of HIF-1α [reviewed in [7]]. In the MIA PaCa-2 pancreatic cell line p38 MAPK activation in response to ischemia resulted in phosphorylation of HIF-1α which inhibited binding to VHL, allowing HIF-1α accumulation [18]. Pharmacological inhibition of p38 in MIA PaCa-2 cells [18], pulmonary fibroblasts [19] and chondrocytes [20] has been shown to abrogate HIF-1α protein expression. Other studies suggest that p38 MAPK promotes formation of HIF-1-p300 complex thereby enhancing HIF-1α transcriptional activity [21].

There have been extensive pre-clinical investigations describing the activity and mechanisms of action of the synthetic glucose analog 2-deoxy-glucose (2-DG) as an anti-cancer agent [22]. Clinically, high dose 2-DG is well tolerated and initial studies suggest it can be safely administered in combination with radiation therapy [23]. 2-DG competes with glucose for intracellular transport via the glucose transporter type 1 (GLUT-1) transporter. In the cell it competes with glucose for hexokinase mediated phosphorylation to form 2-DG-6-phosphate, which is not further metabolized to any significant extent. Multiple mechanisms of action have been proposed for the anti-tumor activity of 2-DG including inhibition of glycolysis with resulting decreases in generation of adenosine triphosphate (ATP) and nicotinamide adenine dinucleotide phosphate and alteration of N-linked glycosylation due to its similarity in structure to mannose*. In vitro*, 2-DG treatment results in oxidative stress [24], activation of endoplasmic reticulum stress and the unfolded protein response [25], and induction of apoptosis and autophagy.

D-allose is a rare sugar with a similar structure to 2-DG produced from D-ribose for which a recent mass production process has been developed [26]. D-allose has been studied in multiple cancer cell line models including ovarian cancer and was demonstrated to have promising anti-tumor proliferation and pro-apoptotic activity [27]. Interestingly, D-allose has also been demonstrated to have anti-inflammatory and anti-oxidant effects [28]. The mechanisms of action of D-allose are still not well understood. Studies in neuroblastoma Neuro2A cells suggest that D-allose competes with D-glucose prior to or at the mitochondrial respiratory chain resulting in a decrease in D-glucose driven ROS production and decrease in ATP synthesis [29].

Resistance to 2-DG has been reported to be mediated by HIF-1α in several cancer cell line models [30]. We therefore hypothesized that p38 MAPK inhibition may increase the efficacy of glucose analogs via suppression of HIF-1α activity. To test this we investigated the activity of 2-DG and D-allose in combination with SB202190, a pharmacological p38 MAPK inhibitor, in a panel of pancreatic and ovarian cancer cell lines. Treatment with SB202190 caused a modest decrease in HIF-1α protein and transcriptional activity in the MIA PaCa2 cells and resulted in enhanced efficacy of both 2-DG and D-allose together and in combination with oxaliplatin in the majority of cell lines investigated. Our study suggests that p38 MAPK inhibition should be investigated further as a strategy to enhance the efficacy of 2-DG or similar metabolic inhibitors.

## Methods

### Materials

Unless otherwise specified, all biochemicals were purchased from Sigma-Aldrich, St. Louis, Missouri.

### Cell culture

MIA PaCa-2, BxPC-3, AsPC-1, OVCAR-3, and SK-OV-3 cells were purchased from American Type Culture Collection (Manassas, VA). The HEY cell line was purchased from Cedarlane (Burlington, ON). The MIA PaCa-2 and BxPC-3, and AsPC-1 cell lines are pancreatic adenocarcinomas. The OVCAR-3, SK-OV-3, and HEY cells are ovarian adenocarcinomas. Cells were grown at 37°C and 5% CO_2_. MIA PaCa-2 and HEY cell lines were cultured in Dulbecco Modified Eagle medium (Cellgro, Manassas, VA) with 10% fetal bovine serum (Thermo Scientific, Rockford, IL) and 4.5 g/L glucose. BxPC-3 and AsPC-1 cell lines were cultured in Roswell Park Memorial Institute medium (RPMI) (Cellgro) with 10% FBS. OVCAR-3 cells were cultured in RPMI (Cellgro) with 15% FBS. SK-OV-3 were cultured in McCoy’s 5A medium (Cellgro) with 10% FBS For hypoxia studies, cells were grown in 1% oxygen, in a humidified, 37°C Ruskinn Invivo2 Hypoxia Workstation (Ruskinn, Pencoed, Bridgend, UK). Cells were tested for mycoplasma contamination using the MycoAlert mycoplasma detection assay kit from Lonza (Basal, Switzerland) and found to be negative. To verify cell line authenticity, genomic DNA was extracted and diluted appropriately in TE buffer (10 mM Tris-CL, pH 7.5, 1 mM EDTA), and submitted to the University of Arizona Genomics Core (Human Origins Gentoyping Lab) for analysis. Autosomal short tandem repeat (STR) typing was conducted across the 13 core STRs in Combined DNA Index System and referenced against allelic peaks in cell lines of previously confirmed genotype. All cell lines were verified as authentic.

### Cell treatments

2-DG and D-allose (Omicron Biochemicals, South Bend, IN) treatments were performed for the concentrations and time points indicated for each experiment. For inhibition of p38 MAPK, a 2 hour pretreatment with 20 μM SB202190 (Calbiochem, San Diego, CA) was performed and continued through the duration of the experiment. Oxaliplatin (Sanofi Aventis, Bridgewater, NJ) and cisplatin treatments were performed at concentrations and time points indicated both alone and in conjunction with 2-DG, D-allose, and or SB202190 treatments.

### Antibodies for western blot

The following antibodies and concentrations for western blotting were used: 1:1000 α-Tubulin (Calbiochem, San Diego, CA), 1:1000 Caspase-3 (Cell Signaling Technology, Danvers, MA), 1:1000 Cleaved poly(ADP-ribose) polymerase (PARP) (Cell Signaling Technology), 1:500 PARP (BD Pharmingen, San Jose, CA) and 1:1000 HIF-1α (Santa Cruz, Dallas, TX). Horseradish peroxidase-conjugated secondary antibodies for mouse and rabbit were used at a dilution of 1:10,000 (Jackson ImmunoResearch, West Grove, PA).

### Lactate assay

Lactate production was analyzed using the L-Lactate Assay Kit I (Eton Bioscience, San Diego, CA) per manufacturer instructions. A standard curve was generated using lactate standards provided in the kit. Lactate concentration of samples was determined by plotting samples on the standard curve. Lactate secreted by the cultured cells was determined by subtracting the lactate concentration of media alone from the lactate concentration of each sample. Intracellular lactate was normalized to protein concentration of the sample using the 660 nm Protein Assay kit (Thermo Scientific).

### Development of a stable-expressing HRE-luciferase MIA PaCa-2 cell line

MIA PaCa-2 cells were stably transfected with a pcDNA3.1 vector expressing luciferase (Invitrogen) controlled by a hypoxia-response element (HRE) motif from the promoter of the VEGF gene (obtained from Dr. Robert Gillies, Lee Moffitt Cancer Center). Transfections were performed using FuGENE HD Transfection Reagent (Roche, Mannheim, Germany) according to manufacturer’s protocol. Treatments of 1200 μg/ml G418 were added to 10%FBS/RPMI cell media 24 hours following transfection to select for stably transfected cells. Fresh media containing antibiotic was added every 2–3 days and selection was performed for 20 days. Following selection, stable cells, defined as HRE-Luc MIA PaCa-2, were pooled based on their baseline luciferase expression as well as the responsiveness of luciferase expression to hypoxia treatments. The pooled cell line demonstrated a 15 fold elevation of HRE expression after 24 hours of growth in 1% O_2_ relative to cells grown in normoxia for 24 hours (data not shown). Cells were maintained in media supplemented with 400 μg/ml G418. Prior to experiments, cells were grown in G418 free media for a minimum of 24 hours.

### Transfection and luciferase assay

Cells were cultured in 96-well or 24-well plates and allowed to reach 80% confluence. Cells were treated with varying concentrations of 2-DG or D-allose, or SB202190 as indicated. Firefly luciferase activity was measured after the times indicated using the Luciferase Assay System kit (Promega, Sunnyvale, CA) in a multi-detection microplate reader (BioTek, Winooski, VT) according to manufacturer’s instructions. Luciferase readings were normalized to the protein concentration of the sample (μg/ml) which was determined using the 660 nm Protein Assay kit (Thermo Scientific). The untreated control group was set to 100%. Biological replicates of each experiment were performed at least three times.

### Q-RT-PCR

Total cellular RNA was extracted using NucleoSpin RNA II total RNA extraction kit (Macherey-Nagel, Duren, Germany). cDNA was generated from 0.5 ug of DNase-free RNA using qScript cDNA Synthesis Kit (Quanta BioSciences, Gaithersburg, MD) per manufacturer’s instructions. Reverse transcription (RT) was performed using a GeneAmp 7300 sequence detection system (PE Biosystems, Foster City, CA). Amplification reactions were set up in a reaction volume of 20 μL by use of the Applied Biosystems TaqMan Univeral PCR Mastermix (Applied Biosystems, Branchburg, NJ). PCR primers and TaqMan probe for LDHA were synthesized and pre-optimized by Applied Biosystems (Assays on Demand) (Foster City, CA). Each reaction contained 1× PCR Mastermix, 900 nmol of each primer, and 250 nmol of the *Fam* probe. Reverse transcription was done at 48°C for 30 minutes, samples incubated for 10 minutes at 95°C and then amplification over 40 cycles at 15 sec at 95°C followed by 1 minute at 60°C. Values were normalized to RPLPO message and quantitated using the delta CT method as described by Perkin-Elmer.

### Western blot analysis

Cells were rinsed with cold PBS and harvested in 50 mM Tris HCl (pH 8.0), 150 mM NaCl, 1% Triton X-100, 2 mM EDTA, 5 mM Na_3_VO_4_, 200 μM NaF, 21 μM leupeptin, 230 nM aprotinin, and 1 mM PMSF. Cell lysate was centrifuged at 10,000 × *g* for 10 minutes at 4°C. Protein concentration of the resulting supernatant was determined using a 660 nm Protein Assay kit (Thermo Scientific). Total cell lysate (30 μg) was boiled for 5 minutes and resolved in acrylamide/bisacrylamide gel by electrophoresis. Proteins were transferred to a polyvinylidene fluoride (PVDF) membrane (Millipore, Billerica, MA) or nitrocellulose membrane (Bio-Rad, Hercules, CA). The membrane was blocked with 5% milk in PBST or TBST and incubated with primary and secondary antibodies according to manufacturer’s recommendations. Reactive bands were visualized by exposure to film using HyGLO Chemiluminescent HRP Detection Reagent (Denville Scientific, Metuchen, NJ) or SuperSignal West Dura Extended Duration Substrate (Thermo Scientific). Blots were stripped in 0.2 M NaOH with shaking for 10 minutes at room temperature.

### MTT cell proliferation assay

The Thiazolyl Blue Tetrazolium Bromide (MTT) assay was used to compare cell proliferation rates. Cells were seeded at a density of 3000 cells/well in a 96-well plate with outer wells left empty for addition of water. After indicated hours of culture, cells were treated with varying concentrations of drug. MTT dye (2 mg/ml) was added to cultures treated as indicated above, and incubated for an additional 4 hours at 37°C. Formazan crystals were dissolved in dimethylsulfoxide (DMSO) for 5 minutes and the plates were read in a spectrophotometer at 540 nm. For studies combining 2-DG or D-allose with platinum analogs, cells were treated with a constant ratio of 2000:1 of each drug, respectively. Results were graphed using GraphPad Prism software and IC_50_ values and combination index values for the IC_50_ concentrations were calculated using CalcuSyn (Biosoft, Great Shelford, UK). Each assay was performed with a minimum of 6 analytical replicates.

### Statistical analysis

Results are expressed as mean ± S.D. Statistics were calculated using GraphPad InStat software (La Jolla, CA). All comparisons to controls were calculated using a one sample t test. Comparisons between treatment groups were analyzed using an unpaired t test.

## Results

### 2-DG and D-allose inhibit lactate accumulation

**Figure 1:**
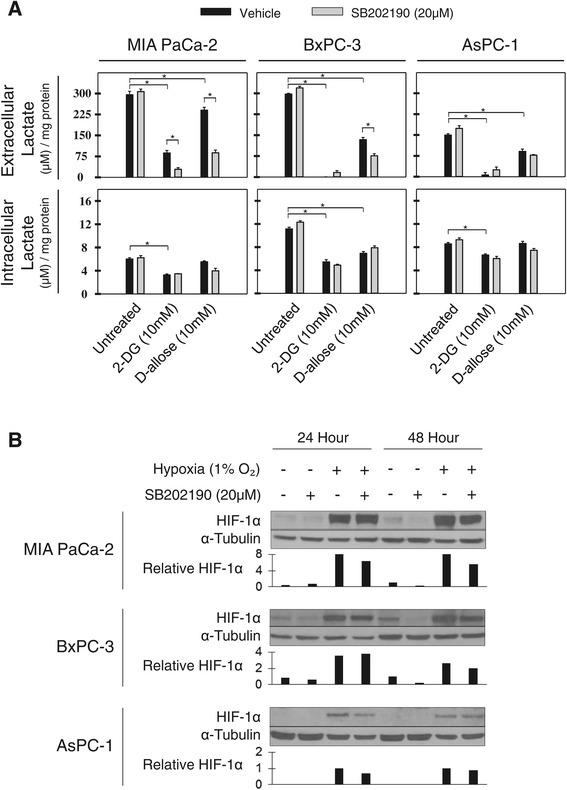
**Lactate production and HIF-1α expression in pancreatic cancer cell lines. A)**. Effect of 2-DG and D-allose alone or in combination with SB202190 following 24 hours of treatment on lactate production measured in cell culture media or in cells. Values are normalized to total cell protein. One representative experiment is shown, n = 3. *p < 0.05 **B)**. Western blot of cells grown in normoxia or hypoxia for 24 and 48 hours treated with SB202190 and analyzed for HIF-1α. Tubulin served as a loading control for protein. HIF-1α band density was quantified using ImageJ software with normalization to α-Tubulin.

To investigate the effect of 2-DG and D-allose treatment on lactate accumulation, we measured intracellular lactate and lactate accumulation in cell culture media in MIA PaCa-2, BxPC-3 and AsPC-1 pancreatic cells grown in normoxia for 24 hours and treated with 10 mM 2-DG or D-allose alone (black bars), or in combination with 20 μM SB202190 (grey bars) (Figure 1A). In the MIA PaCA-2 cell line 2-DG and D-allose inhibited extracellular lactate accumulation in the media, with 2-DG showing the greatest effect at equalmolar concentrations. SB202190 further reduced lactate accumulation in combination with both glucose analogs in the MIA PaCA-2 cell line, but had mixed effects in the BxPC-3 and AsPC-1 cells. Intracellular lactate levels reflect both production and export of lactate which is influenced by expression and activity of monocarboxylate transporters. There was a trend for decreased intracellular accumulation of lactate following treatment with 2-DG and D-allose, with no further decrease in combination with SB202190. However, statistical significance was only observed in the vehicle control compared to 2-DG treated cell lines. Together, these results suggest that similar to 2-DG, D-allose has a moderate impact on cellular metabolism that is cell line dependent.

### SB202190 treatment results in a modest inhibition of HIF-1α protein accumulation

Because HIF-1α regulates glycolytic activity, we investigated HIF-1α protein in MIA PaCa-2, BxPC-3, and AsPC-1 grown in normoxia or hypoxia (1% O_2_) treated with vehicle alone or SB202190 (Figure 1B). When cell lines were grown in normoxia, constitutive levels of HIF-1α were observed in MIA PaCa-2 and BxPC-3 cells but not in AsPC-1 cells. This observation was consistent with a higher level of lactate produced in normoxia in the MIA PaCa-2 and BxPC-3 compared to the AsPC-1 cells (Figure 1A). After 48 hours of treatment in normoxia, SB202190 treatment diminished HIF-1α protein accumulating in MIA PaCA-2 and BxPC-3 cells. When the same cells were grown in hypoxia (1% O_2_), the amount of hypoxia-induced HIF-1α was only mildly decreased after 48 hours of treatment with SB202190.

### SB202190 inhibits HRE-luciferase reporter activity in MIA PaCa2 cells alone and in combination with glucose analogs

**Figure 2:**
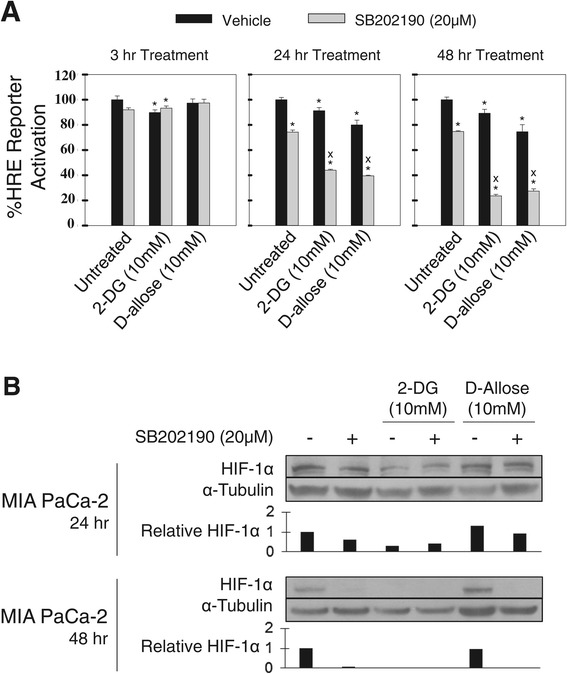
**HIF-1α transcriptional activity is modulated by SB202190. A)**. MIA PaCa-2 cells were stably transfected with a HRE-luciferase reporter and treated for 3 24, and 48 hours with 2-DG or D-allose alone and in combination with SB202190. Luciferase activity was normalized to total protein. One representative experiment is shown, n = 3. *Indicates a p < 0.05 relative to vehicle control. X indicates a p < 0.01 relative to SB202190 treated control. **B)**. Effect of glucose analogs alone and in combination with SB202190 after 24 and 48 hours in MIA PaCa-2 cells measured by Western blot analysis of HIF-1α. Tubulin was used as a loading control. Quantification of HIF-1α band density was performed using ImageJ software with normalization to α-Tubulin.

Using MIA PaCa-2 cells stably transfected with a HRE reporter, we investigated the ability of 2-DG and D-allose to decrease HIF-1α transcriptional activity. Although both analogues significantly decreased activity, a greater effect was observed with D-allose at both 24 and 48 hours compared to 2-DG following treatment (black bars) (Figure 2A). We also investigated the ability of SB202190 to modulate HRE activity (grey bars). A modest but statistically significant (p < 0.0001) decrease in reporter activity after 24 and 48 hours of treatment was observed. When 2-DG or D-allose were combined with SB202190, a greater decrease in reporter activity was observed at 24 and 48 hours (p < 0.0001) compared to either glucose analog alone.

### LDHA gene expression is inhibited following treatment with glucose analogs and SB202190

We investigated whether treatment with glucose analogs alone or in combination with SB202190 resulted in changes in gene expression of the HIF-1 α regulated gene LDHA using qRT-PCR (data not shown). SB202190 treatment decreased LDHA gene expression in normoxia and hypoxia, but changes were only statistically significant under hypoxia conditions (p < 0.05). 2-DG alone showed similar results, with a small decrease in normoxia (p > 0.05), but a greater decrease in hypoxia (p < 0.05). D-allose alone did not impact LDHA gene expression. SB202190 abrogated gene expression of LDHA when combined with 2-DG (p < 0.0001) or with D-allose (p < 0.05) in hypoxia. A similar trend was observed in normoxia, but was not significant.

### SB202190 inhibits HIF-1α accumulation in MIA PaCa2 cells in combination with glucose analogs

Using the wild-type MIA PaCa-2 cell line we also investigated the effect of SB202190 on HIF-1α accumulation in combination with 2-DG and D-allose after 24 and 48 hours of treatment (Figure 2B). Treatment with 2-DG and D-allose alone caused a time dependent decrease in HIF-1α accumulation which was further inhibited in combination with SB202190. It should be noted that the decrease in HIF-1α may be an acute effect of treatment given the prior reports that one mechanism of resistance to 2-DG is upregulation of HIF-1α [30]. These results suggest that SB202190 may provide additive or synergistic activity with glucose analog treatment for tumor cells growing in an oxygenated microenvironment. To investigate this hypothesis, proliferation studies were conducted only in normoxia.

### SB202190 enhances 2-DG and D-allose mediated PARP cleavage

**Figure 3:**
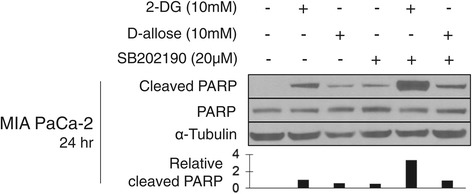
**SB202190 enhances cleavage of PARP when combined with glucose analogs.** Western blot analysis of whole cell lysates from cells treated for 24 hours with 2-DG or D-allose alone or in combination with SB202190. Lysates were probed for cleaved and total PARP. Tubulin served as a loading control for protein. Cleaved PARP band density was quantified using ImageJ software with normalization to α-Tubulin. One representative experiment is shown, n = 3.

To investigate if p38 MAPK inhibition sensitizes cancer cells to apoptosis we pre-treated MIA PaCa-2 cells with SB202190 for 2 hours and then added 10 mM 2-DG or 10 mM D-allose (with no washout of SB202190) for 24 hours. Western blot analysis of whole cell lysates was performed and the blots probed for cleaved and total PARP (Figure 3). Cleaved PARP levels, indicative of apoptosis, were observed in cells treated with both 2-DG and D-allose, although 2-DG caused greater PARP cleavage than D-allose. SB202190 alone also resulted in a small level of PARP cleavage and enhanced PARP cleavage in combination with 2-DG and D-allose. Similar results were observed at 48 hours (data not shown).

### Comparison of anti-proliferative activity of 2-DG and D-allose

**Figure 4:**
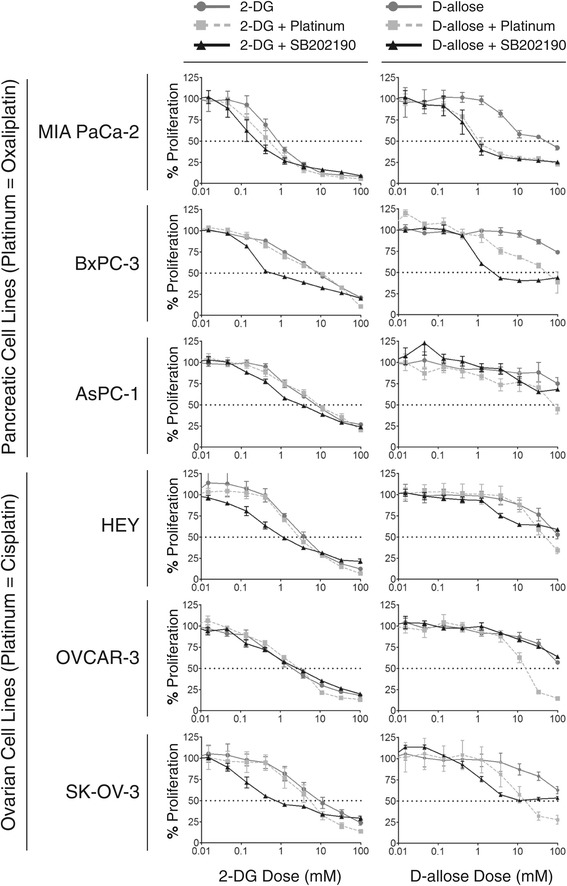
**Effect of dosing regimens on the proliferation of pancreatic and ovarian cell lines following 48 hours of treatment.** Cells were treated with increasing doses of 2-DG or D-allose alone or in combination with oxaliplatin (pancreatic cell lines), cisplatin (ovarian cell lines) and/or SB202190. Cell proliferation was measured using the MTT assay. The ratio of 2-DG or D-allose to the platinum analogs was 2000:1 for all doses, respectively. One representative experiment is shown, n = 6.

To compare the anti-proliferative activity of 2-DG to D-allose we used both pancreatic and ovarian cancer cell line models. Cell lines were treated with increasing doses of 2-DG or D-allose for 48 hours and analyzed proliferation using the MTT assay (Figure 4). 2-DG showed marked anti-proliferative activity with IC_50_ values ranging between 1.45 to 13.34 mM (Table 1). However, D-allose did not significantly alter tumor proliferation rates, with the most sensitive cell line being the MIA PaCa2 cell line having an IC_50_ value of 53.25 mM (Table 2). A study by Siu et al. [27] demonstrated D-allose had growth inhibitory effects in the OVCAR-3 cell line (50 mM) after 5 days of treatment. It is possible that longer treatment duration may result in greater activity, but due to the fast growth rate of many of the cell lines in our panel we limited treatment duration to 48 hours.

**Table 1:** **Anti-proliferative activity (IC**_**50**_ **values) of 2-DG alone and in combination with platinum analogs and/or SB202190**

**Cell line**	**2-DG (mM)**	**Platinum (μM)**	**2-DG + Platinum**			**2-DG + 20uM SB202190 (mM)**	**Platinum + 20uM SB202190 (uM)**	**2-DG + 20 μM SB202190 + Platinum**	
			**2-DG (mM)**	**Platinum (μM)**	**CI at IC50**			**2-DG (mM)**	**Platinum (μM)**
**MIA PaCa-2**	1.45	3.37	0.95	0.48	0.796	0.48	19.72	0.24	0.12
**BxPC-3**	10.80	28.13	8.12	4.06	0.896	0.60	32.50	1.23	0.61
**ASPC-1**	11.47	>50	10.55	5.25	0.954	4.90	>50	5.77	2.88
**OVCAR-3**	3.39	6.93	3.39	1.69	1.243	3.9	26.45	4.1	2.05
**HEY**	10.17	32.90	9.88	4.94	1.122	2.63	50>	2.44	1.22
**SK-OV-3**	13.34	11.53	6.46	3.23	0.764	1.42	21.05	2.01	1.01

IC_50_ values represent the level of drug that inhibited cell growth by 50% following 48 hours of treatment. Combination index (CI) values are defined as: <0.1 = very strong synergy, 0.1-0.3 = strong synergism, 0.3-0.7 = synergism, 0.7 – 0.85 = moderate synergism, 0.85-0.9 = slight synergism, 0.9-1.1 = nearly additive, 1.1-1.45 slight to moderate antagonism.

**Table 2:** **Anti-proliferative activity (IC**_**50**_ **values) of D-allose alone and in combination with platinum analogs and/or SB202190**

			**D-allose + Platinum**			**D-allose + 20 μM SB202190 (mM)**	**D-allose + 20 μM SB202190 + Platinum**	
**Cell line**	**D-allose (mM)**	**Platinum (μM)**	**D-allose (mM)**	**Platinum (μM)**	**CI at IC50**		**D-allose (mM)**	**Platinum (μM)**
**MIA PaCa-2**	53.25	3.37	4.16	0.95	0.696	3.18	1.98	0.99
**BxPC-3**	>100	28.13	43.3	21.65	0.787	4.09	8.75	4.38
**ASPC-1**	>100	>50	>100	>50	n/c	74.11	>100	>50
**OVCAR-3**	>100	6.93	15.84	7.92	1.171	>100	51.7	25.85
**HEY**	>100	32.90	52.68	26.34	1.002	94.17	53.01	26.5
**SK-OV-3**	>100	11.53	18.55	9.27	0.857	10.82	9.74	4.87

IC_50_ values represent the level of drug that inhibited cell growth by 50% following 48 hours of treatment. Combination index (CI) values are defined as: <0.1 = very strong synergy, 0.1-0.3 = strong synergism, 0.3-0.7 = synergism, 0.7 – 0.85 = moderate synergism, 0.85-0.9 = slight synergism, 0.9-1.1 = nearly additive, 1.1-1.45 slight to moderate antagonism.

To investigate whether the combination of 2-DG or D-allose with platinum analogs was more efficacious than combination with p38 MAPK inhibition by SB202190, we performed MTT analysis of 2-DG and D-allose in combination with oxaliplatin (in pancreatic cell lines) or cisplatin (in ovarian cell lines) following treatment for 48 hours (Figure 4, and Tables 1 and 2). Using the median effect method as described by T-C Chou and P Talalay [for a recent review of this method the reader is referred to [31]], we calculated the combination index (CI) at the IC_50_ value of each drug using CalcuSyn software (Biosoft, Ferguson, MO). The addition of 2-DG to platinum agent resulted in additive to moderate synergy in MIA PaCA-2 (CI 0.796), BxPC-3 (CI = 0.896), AsPC-1 (CI = 0.954), and SK-OV-3 (CI = 0.764). The addition of D-allose to platinum agent also resulted in additive to moderate synergy in the MIA PaCa-2 (CI = 0.696), BxPC-3 (CI = 0.787), and SK-OV-3 (CI = 0.857) cells. The ASPC-1 cells, which are inherently resistant to oxaliplatin and remained inherently resistant to both compounds with IC_50_ values being above feasible dose concentrations. Because SB202190 was kept at a constant dose during cell treatments, combination indices were not calculated. Overall, we observed greater growth inhibition when 2-DG or D-allose was combined with SB202190 than when combined with platinum compounds.

### SB202190 sensitizes cell lines to treatment with 2-DG and D-allose

To investigate the impact of p38 inhibition on 2-DG and D-allose anti-proliferative activity, we pre-treated cells for 2 hours with 20 μM SB202190 and then added 2-DG or D-allose for an additional 48 hours (Figure 4). Treatment with 20 μM SB202190 alone for 50 hours did not cause significant inhibition of cell proliferation (the % change in absorbance values of treated compared to vehicle control ranged from 71.69 to 97.18%; (data not shown). The combination of 2-DG and SB202190 resulted in a decreased IC_50_ value for all cell lines investigated except OVCAR-3 cells. BxPC-3 cells showed the greatest sensitivity to this combination with the IC_50_ value dropping from <10.8 mM to 0.60 mM. The combination of D-allose and SB202190 resulted in enhanced anti-proliferative activity in the MIA PaCa2, BxPC-3 and SK-OV-3 cell lines, with IC_50_ values <11 mM.

### SB202190 is antagonistic with platinum compounds alone, but enhance anti-proliferative activity when combined with platinum plus glucose analogs

We also investigated the activity of platinum compounds in combination with SB202190 (Figure 4, Tables 1 and 2). We observed combined treatment with SB202190 and platinum agents resulted in reduced anti-proliferative activity, with markedly higher (2 fold or higher) IC_50_ values in both pancreatic and ovarian cancer cell lines. However, when cells were pre-treated with SB202190 followed by addition of platinum agent and 2-DG, enhanced anti-proliferative activity was observed for all cell lines investigated, including the AsPC-1 cell line which was highly resistant to oxaliplatin monotherapy. Pre-treatment with SB202190 followed by addition of platinum and D-allose also resulted in decreased IC_50_ values in MIA PaCa-2, BxPC-3, and SK-OV-3 cell lines.

## Discussion

Pancreatic cancer and ovarian cancer are both lethal diseases that are molecularly complex. Clinical results with molecularly targeted agents in these malignancies have in general been disappointing and current treatment strategies still rely heavily on chemotherapy and radiation. Gemcitabine remains the first line standard of care in pancreatic cancer and cisplatin/taxol is one of the primary standard of care treatments for ovarian cancer. Oxaliplatin is used as second line treatment in combination with a fluoropyrimidine (5-FU/leucovorin or capecitabine) in patients with advanced or metastatic pancreatic cancer who have good performance status [32]. New treatment strategies are needed that can be safely combined with chemotherapy and/ or radiation to overcome resistance.

Aerobic glycolysis is a hallmark of tumor cells resulting in the conversion of pyruvic acid to lactic acid in oxygenated conditions. Lactic acid is rapidly exported from cells by monocarboxylate transporters to maintain a neutral intracellular pH [33]. Thus, accumulation of extracellular lactate is correlated with metabolic activity. Targeting these tumor phenotypes that are glycolytically active using the glucose analog 2-DG has been extensively investigated pre-clinically in a wide variety of tumor types. Our results, summarized in Tables 1 and 2, demonstrate marked differences in response to glucose analog treatment across cell lines. This may be a partially due to differences in proliferation rates of the cells (for example MIA PaCa-2 cells grow faster than BxPC-3 or AsPC-1 cells). However, proliferation rate is not the only factor influencing sensitivity to glucose analogs as demonstrated by the HEY ovarian cancer cell line which grows much faster than the OVCAR-3 cells, but had a higher IC50 value at 48 hours (10.17 vs 3.39 mM, respectively).

2-DG is clinically well tolerated [24], suggesting that pre-clinical findings with standard of care chemotherapy could be translated to the clinic. Preclinical studies have shown enhanced anti-tumor activity in pancreatic models with a variety of drugs including gemcitabine [34]. Cisplatin has demonstrated enhanced cytotoxicity in combination with 2-DG in head and neck cancer cells and in lung cancer cell lines [35,36]. Cisplatin-resistant cancer cell lines with decreased hexokinase levels have been shown to be more sensitive to the combined activity of cisplatin with 2-DG or 2-fluorodeoxyglucose [31]. Our results also demonstrate enhanced anti-tumor activity of 2-DG in combination with cisplatin in ovarian cancer cell lines and oxaliplatin in pancreatic cancer cell lines. However, we did not investigate the correlation of hexokinase activity with this effect. HIF-1α is often associated with resistance to chemotherapy including oxaliplatin [37] and cisplatin [38], and has also been shown to mediate resistance to 2-DG [29]. Pancreatic and ovarian cancers have high expression of HIF-1α [39,40]. Activation of the p38 MAPK pathway has been associated with cisplatin resistance, although the effect of p38 MAPK inhibition is highly variable across cell lines [41]. Our studies demonstrated enhanced anti-proliferative activity in some cell lines when glucose and platinum analogs were combined with a p38 MAPK inhibitor. This combined activity may be partially due to abrogation of HIF-1α transcriptional activity mediated by p38 MAPK inhibition. However, further mechanistic studies are warranted to fully understand the context in which p38 MAPK may be beneficial in combination with glucose analogs.

The tumor microenvironment is characterized not only by low oxygen levels, but oxidative conditions and inflammation which can all contribute to activation of the p38 MAPK signaling pathway. The p38 MAPK pathway is involved in many cellular functions including proliferation, cellular differentiation, and apoptosis. It has also been linked with DNA repair and regulation of p-glycoprotein. The level and extent of p38 MAPK signal in addition to the molecular context of expression are all important in determining the function of p38 MAPK [17]. Our results suggest that p38 MAPK inhibition in glycolytically active tumors expressing high levels of HIF-1α, or in oxidized cells with increased HIF-1α activity should be further studied. However, it should be appreciated that given the many biological roles of p38 MAPK there are probably multiple mechanisms by which p38 MAPK inhibitors may modulate response to drug therapy in addition to HIF-1α regulation. It should also be noted that in situations where cell proliferation is regulated through growth factor signaling via the PI3K/AKT pathway as opposed to stress signaling via ROS, inhibition of the p38 MAPK may have little therapeutic benefit as recently shown in a study by Cheng et al. [42].

D-allose has shown promising anti-proliferative and pro-apoptotic activity in other cancer cell line models, but to our knowledge the anti-tumor activity of D-allose has not been studied in pancreatic cancer models. In the current study we observed moderate single agent D-allose anti-proliferative activity in the MIA PaCa-2 cell line and promising synergistic activity with oxaliplatin in MIA PaCa-2, BxPC-3, and SK-OV-3 cell lines. Similarly, D-allose had anti-proliferative activity in these same cells lines when pre-treated with SB202190. Notably, the MIA PaCa-2 and BxPC-3 cell lines accumulate greater extracellular lactate than the AsPC-1 cell line that was resistant to D-allose monotherapy or combined drug treatments. Interestingly, D-allose resulted in greater inhibition of HRE-luciferase activation compared to 2-DG although it was less potent at inhibiting cell proliferation. Together, these observations suggest that D-allose may have distinct mechanistic differences as compared to 2-DG.

Cisplatin is associated with a high incidence of nephrotoxicity. D-allose has been shown to inhibit the production of inflammatory mediators of nephrotoxicity including TNF-alpha and renal monocyte chemoattractant protein-1 and to protect against cisplatin induced renal tubular injury in mice [43]. Our results demonstrate that D-allose does not inhibit the anti-proliferative activity of oxaliplatin or cisplatin, and even enhances platinum mediated anti-proliferative activity in selected cell lines. Therefore, D-allose may not only act as a chemosesitizer but also have renal protective effects in combination with cisplatin.

## Conclusions

Tumor cells develop multiple adaptive survival mechanisms including metabolic adaptations. Combination cancer therapies that target cancer metabolism via modulation of HIF-1α are attractive due to the central role HIF-1α plays in cancer metabolism and drug resistance. Our studies provide the rationale to investigate glucose analogs, which target metabolism, in combination with p38 MAPK inhibitors, which modulate HIF-1α. However, the combination of only p38 MAPK inhibitors with platinum agents should be conducted with caution.

## Abbreviations

2-DG: 2-deoxy-glucose
HIF-1α: Hypoxia inducible factor-1 alpha
PARP: Cleaved poly (ADP-ribose) polymerase
MAPK: Mitogen-activated protein kinase
LDHA: Lactate dehydrogenase A
VHL: Von-Hippel-Lindau
PI3K/AKT: Phosphoinositide 3-kinase/ protein kinase B
ERK: Extracellular signal-regulated kinases
GLUT-1: Glucose transporter type 1
ATP: Adenosine triphosphate
RPMI: Roswell Park Memorial Institute medium
HRE: Hypoxia-response element
MTT: Thiazolyl Blue Tetrazolium Bromide
CI: Combination index
IC_50_: Inhibitory concentration 50
